# Spatiotemporal patterns and climate influences on leptospirosis in Sri Lanka from 2009 to 2024

**DOI:** 10.1186/s12879-026-12533-1

**Published:** 2026-01-12

**Authors:** Nayana Gunathilaka, Deshaka Jayakody, Saranga Erathna

**Affiliations:** Department of Parasitology, Faculty of Medicine, University of Kelaniya, Ragama, Sri Lanka

**Keywords:** Leptospirosis, Spatiotemporal analysis, Climate variability, GAMLSS, Moran’s i epidemiology, Sri lanka

## Abstract

**Background:**

Leptospirosis remains a major public health concern in Sri Lanka, a country with a tropical climate conducive to transmission. Despite ongoing surveillance, there is limited evidence on the spatial and climatic determinants driving long-term disease dynamics. This study aimed to investigate the spatiotemporal distribution and climatic sensitivity of leptospirosis from 2009 to 2024 using advanced statistical modelling.

**Methods:**

District-level monthly leptospirosis case data for the period 2009–2024 were obtained from the Epidemiology Unit of the Ministry of Health, Sri Lanka. Corresponding monthly district-level climatic variables, including total rainfall, mean temperature, minimum temperature, maximum temperature, and mean relative humidity, were retrieved from the NASA POWER satellite dataset. Associations between climatic variables and leptospirosis incidence, as well as spatial heterogeneity in case distribution, were assessed using a Generalized Additive Model for Location, Scale, and Shape with a Zero-Adjusted Gamma distribution, while spatial clustering and autocorrelation were examined using Moran’s I.

**Results:**

A total of 81,629 confirmed cases of leptospirosis were recorded during the study period. The ZAGA-GAMLSS model identified several climatic, spatial, and temporal predictors that were significantly associated with district-level incidence. Relative humidity and maximum temperature showed immediate negative associations with incidence, while humidity, mean temperature, and rainfall demonstrated positive lag-dependent effects at 1–3 months. In contrast, maximum and minimum temperatures exhibited predominantly negative association at 3-month lag. Spatial heterogeneity was evident in both incidence rates and zero-inflation, and temporal dependence was detected at 1 and 12-month lags. Higher relative humidity three months earlier was linked to more stable leptospirosis incidence rates across districts. Spatial analysis further revealed significant clustering, with hotspots identified in the districts of Ratnapura, Galle, Matara, and Hambantota, while Colombo was identified as a spatial outlier.

**Conclusions:**

Leptospirosis in Sri Lanka exhibits distinct spatiotemporal patterns influenced by climatic variability, with elevated risk following monsoon rains in the south‑western part of the country. Climate‑sensitive modelling, as demonstrated in this study, supports the integration of meteorological surveillance into early warning and response systems to enhance leptospirosis control, particularly in identified hotspot regions.

**Supplementary Information:**

The online version contains supplementary material available at 10.1186/s12879-026-12533-1.

## Background

Leptospirosis is a neglected but globally significant zoonotic disease caused by pathogenic spirochetes of the genus *Leptospira* [1]. The disease is transmitted to humans primarily through direct or indirect contact with the urine of infected animals, with rodents serving as the principal reservoirs [2]. It has a wide range of clinical manifestations, from mild febrile illness to severe forms characterized by multi-organ involvement, such as Weil’s disease and pulmonary haemorrhage syndrome, leading to high case fatality rates if untreated [3]. The World Health Organization (WHO) has identified leptospirosis as an emerging infectious disease of global importance, particularly in tropical regions with heavy rainfall and frequent flooding, where environmental conditions enhance bacterial survival and human exposure [4].

In Sri Lanka, leptospirosis was declared a notifiable disease in 2008 due to its increasing public health burden [5]. Since then, the country has reported recurrent outbreaks with considerable morbidity and mortality, particularly in farming communities and areas prone to flooding [6]. Epidemiological surveillance data reveal that leptospirosis incidence in Sri Lanka is not evenly distributed, with certain districts such as Kegalle, Kandy, Gampaha, Ratnapura, and Kalutara consistently reporting higher numbers of cases [7]. Moreover, disease dynamics have shown temporal fluctuations over the past decade, with distinct peaks following monsoonal rains and extreme weather events. These patterns suggest a strong ecological and climatic influence on disease transmission.

Climatic variables, including rainfall, temperature, humidity, and flooding, have been shown to significantly influence leptospirosis occurrence by affecting the survival of *Leptospira* in the environment and altering patterns of human exposure through agriculture, occupational activities, and displacement during floods [8, 9]. Sri Lanka, being a tropical island heavily influenced by the South-West and North-East monsoons, is highly vulnerable to climate variability, which directly impacts the epidemiology of many infectious diseases, including leptospirosis [10]. Despite this, there has been limited systematic analysis of how climate variability over the past 15 years has influenced the spatiotemporal distribution of leptospirosis across districts in the country.

Several Sri Lankan studies have focused on outbreak investigations and short-term epidemiological assessments [7, 11, 12], as well as examined trends in leptospirosis and their relationship to climatic influences [6, 13, 14]. However, these analyses were restricted in scope, focusing primarily on selected districts or climate zones rather than all 25 districts, covered shorter time frames, relied primarily on descriptive statistics or conventional time-series models, and did not incorporate advanced approaches to address zero inflation, overdispersion, or spatial autocorrelation. In contrast, the present study synthesizes 16 years of national surveillance data at the district level and applies a Zero‑Adjusted Gamma GAMLSS framework, enabling the quantification of non‑linear climatic effects and geographic heterogeneity in leptospirosis transmission.

The primary objective of this study is to examine the associations between climatic parameters and the spatiotemporal dynamics of leptospirosis incidence in Sri Lanka. Specifically, the study aims to: (i) characterize temporal trends in leptospirosis incidence; (ii) examine the spatial distribution and district-level heterogeneity of disease burden and identify leptospirosis hotspots; and (iii) quantify the associations between key climatic variables and leptospirosis transmission. The findings are expected to provide an evidence base to strengthen disease surveillance, inform resource allocation, and support the development of integrated, climate-informed public health interventions for leptospirosis control in Sri Lanka.

## Method

### Study design

This study employed a retrospective ecological design to investigate the spatiotemporal and climatic determinants of leptospirosis in Sri Lanka over a 16‑year period (2009–2024). District-level monthly incidence rates served as the unit of analysis, allowing for comparison across all 25 administrative districts. The design integrated epidemiological surveillance data with climatic variables and applied advanced statistical modeling to quantify temporal trends, spatial heterogeneity, and climate‑sensitive associations.

### Study area

Sri Lanka is a tropical island located in the Indian Ocean, covering an area of approximately 65,610 km². The island’s topography is characterized by a central mountainous region that rises to over 2,500 m and is surrounded by low-lying coastal plains [13]. The rainfall pattern in the country is primarily influenced by two seasonal monsoons, the Southwest Monsoon (May–September) and the Northeast Monsoon (December–February) [14]. Rainfall distribution varies considerably across the island, ranging from about 900 mm in the southeastern lowlands to more than 5,500 mm in the southwestern highlands [15]. Temperature also exhibits notable spatial variation, ranging from approximately 17 °C in the central highlands to around 33 °C in the coastal regions, which contributes to the formation of distinct climatic and ecological zones [14].

### Data sources

Leptospirosis case data were obtained from the Epidemiology Unit, Ministry of Health, Sri Lanka, which maintains the national notifiable disease surveillance system [16]. Weekly Epidemiological Reports served as the primary data sources, capturing both laboratory-confirmed and clinically suspected cases based on standard case definitions. District-level monthly case counts were extracted for the 16-year study period (2009–2024). Climate data: mean, maximum, and minimum temperatures; total rainfall; and mean relative humidity, were obtained monthly using the nasapower package in R (version 4.5.2), which accesses NASA’s POWER (Prediction of Worldwide Energy Resources) satellite database [17].

### Case definition

The Ministry of Health defines a suspected case of leptospirosis as a person with acute febrile illness of less than 14 days’ duration with headache, myalgia, and prostration associated with any of the following: conjunctival suffusion, meningism, jaundice, oliguria or anuria, haemorrhages, cardiac arrhythmia or failure, or skin rash which should be notified to the Epidemiology Unit, Sri Lanka [18]. Whenever possible, clinical suspicion of leptospirosis could be supported by appropriate laboratory investigations, including the microscopic agglutination test (MAT) for a high or rising antibody titre, enzyme-linked immunosorbent assay (ELISA), and antigen detection using polymerase chain reaction (PCR), performed at the Medical Research Institute, Sri Lanka, upon request [19].

## Data analysis

### Incidence rate calculation

The incidence rate of leptospirosis was calculated using the formula:

$$\:\mathrm{I}\mathrm{n}\mathrm{c}\mathrm{i}\mathrm{d}\mathrm{e}\mathrm{n}\mathrm{c}\mathrm{e}\:\mathrm{r}\mathrm{a}\mathrm{t}\mathrm{e}=\frac{\mathrm{T}\mathrm{o}\mathrm{t}\mathrm{a}\mathrm{l}\:\mathrm{N}\mathrm{u}\mathrm{m}\mathrm{b}\mathrm{e}\mathrm{r}\:\mathrm{o}\mathrm{f}\:\mathrm{n}\mathrm{e}\mathrm{w}\:\mathrm{c}\mathrm{a}\mathrm{s}\mathrm{e}\mathrm{s}}{\mathrm{T}\mathrm{o}\mathrm{t}\mathrm{a}\mathrm{l}\:\mathrm{n}\mathrm{u}\mathrm{m}\mathrm{b}\mathrm{e}\mathrm{r}\:\mathrm{a}\mathrm{t}\:\mathrm{r}\mathrm{i}\mathrm{s}\mathrm{k}}\:\times\:100\:000$$

In here, the total number of new cases represented the district-level reported cases of leptospirosis during the study period, while the mid-year population was used as the denominator to represent the population at risk. The incidence rate was expressed per 100,000 population to allow for comparison across districts and time periods.

### Confidence interval

The 95% confidence interval for the incidence rate was calculated using the following formula,

$$\:95\boldsymbol{\%}\:\boldsymbol{C}\boldsymbol{I}\:\boldsymbol{o}\boldsymbol{f}\:\boldsymbol{i}\boldsymbol{n}\boldsymbol{c}\boldsymbol{i}\boldsymbol{d}\boldsymbol{e}\boldsymbol{n}\boldsymbol{c}\boldsymbol{e}\:\boldsymbol{r}\boldsymbol{a}\boldsymbol{t}\boldsymbol{e}=\boldsymbol{p}\:\pm\:1.96\:\sqrt{\frac{\boldsymbol{p}\boldsymbol{*}(1-\boldsymbol{p})}{\boldsymbol{r}\boldsymbol{i}\boldsymbol{s}\boldsymbol{k}\:\boldsymbol{p}\boldsymbol{o}\boldsymbol{p}\boldsymbol{u}\boldsymbol{l}\boldsymbol{a}\boldsymbol{t}\boldsymbol{i}\boldsymbol{o}\boldsymbol{n}}}$$

### Modeling of leptospirosis case incidence

Leptospirosis incidence rates were analyzed using a Zero-Adjusted Gamma (ZAGA) distribution within the Generalized Additive Model for Location, Scale, and Shape (GAMLSS) framework [20]. This modeling approach was chosen to effectively address the substantial zero inflation in the Leptospirosis incidence data, with 15% of reported months having zero incidence rates. Model selection was performed by comparing five candidate ZAGA–GAMLSS models with different combinations of predictor variables using Akaike Information Criterion (AIC) and Bayesian Information Criterion (BIC) values (Supplementary Material 1).

In the selected model, the incidence rate was specified as the response variable. Environmental parameters, including mean relative humidity, mean temperature, maximum temperature, minimum temperature, and total rainfall, were included as predictor variables. To account for delayed environmental effects, these predictors were incorporated with lag periods of up to three months (lag 1, lag 2, and lag 3). Spatial effects were captured by including the latitude and longitude of district centroids as predictor variables, while temporal variation was addressed through the inclusion of year and month. To account for temporal autocorrelation in the outcome, lagged incidence rates at 1-month and 12-month intervals were included as additional predictors. The ZAGA spatial GAMLSS model consists of three linked equations that model: the expected incidence rate (µ), the dispersion parameter (α), and the zero-inflation probability (ν),

each as smooth functions of the predictor variables.

(1) Expected Incidence Rate Model

$$\begin{aligned} \:{\mathrm{log}}\left( {\mu {\:_i}} \right) = & \beta {\:_0} + \:{f_1}\left( {mean\:relative\:humidit{y_i}} \right) \\ & + \:{f_2}\left( {mean\:relative\:humidity\:lag\:{1_i}} \right) \\ & + \:{f_3}\left( {mean\:relative\:humidity\:lag\:{2_i}} \right) \\ & + \:{f_4}\left( {mean\:relative\:humidity\:lag\:{3_i}} \right) \\ & + \:{f_5}\left( {mean\:temperatur{e_i}} \right) \\ & + \:{f_6}\left( {mean\:temperature\:lag\:{1_i}} \right) \\ & + \:{f_7}\left( {mean\:temperature\:lag\:{2_i}} \right) \\ & + \:{f_8}\left( {mean\:temperature\:lag\:{3_i}} \right) \\ & + \:{f_9}\left( {maximum\:temperatur{e_i}} \right) \\ & + \:{f_{10}}\left( {maximum\:temperature\:lag\:{1_i}} \right) \\ & + \:{f_{11}}\left( {maximum\:temperature\:lag\:{2_i}} \right) \\ & + \:{f_{12}}\left( {maximum\:temperature\:lag\:{3_i}} \right) \\ & + \:{f_{13}}\left( {minimum\:temperatur{e_i}} \right) \\ & + \:{f_{14}}\left( {minimum\:temperature\:lag\:{1_i}} \right) \\ & + \:{f_{15}}\left( {minimum\:temperature\:lag\:{2_i}} \right) \\ & + \:{f_{16}}\left( {minimum\:temperature\:lag\:{3_i}} \right) \\ & + \:{f_{17}}\left( {total\:rainfal{l_i}} \right) \\ & + \:{f_{18}}\left( {total\:rainfall\:lag\:{1_i}} \right) \\ & + \:{f_{19}}\left( {total\:rainfall\:lag\:{2_i}} \right) \\ & + \:{f_{20}}\left( {total\:rainfall\:lag\:{3_i}} \right) \\ & + \:{f_{21}}\left( {yea{r_i}} \right) + \:{f_{22}}\left( {mont{h_i}} \right) \\ & + \:{f_{23}}\left( {latitud{e_i}} \right) + \:{f_{24}}\left( {longitud{e_i}} \right) \\ & + \:{f_{25}}\left( {\:incidecnce\:rate\:lag\:{1_i}} \right) \\ & + {f_{26}}\left( {\:incidecnce\:rate\:lag\:{{12}_i}} \right) \\ \end{aligned} $$

(2) Dispersion Model

$$\begin{aligned} \:{\mathrm{log}}\left( {\alpha {\:_i}} \right)= &\:\gamma {\:_0} + \:{f_1}\left( {mean\:relative\:humidit{y_i}} \right) \\ & + \:{f_2}\left( {mean\:relative\:humidity\:lag\:{1_i}} \right) \\ & + \:{f_3}\left( {mean\:relative\:humidity\:lag\:{2_i}} \right) \\ & + \:{f_4}\left( {mean\:relative\:humidity\:lag\:{3_i}} \right) \\ & + \:{f_5}\left( {mean\:temperatur{e_i}} \right) \\ & + \:{f_6}\left( {mean\:temperature\:lag\:{1_i}} \right) \\ & + \:{f_7}\left( {mean\:temperature\:lag\:{2_i}} \right) \\ & + \:{f_8}\left( {mean\:temperature\:lag\:{3_i}} \right) \\ & + \:{f_9}\left( {maximum\:temperatur{e_i}} \right) \\ & + \:{f_{10}}\left( {maximum\:temperature\:lag\:{1_i}} \right) \\ & + \:{f_{11}}\left( {maximum\:temperature\:lag\:{2_i}} \right) \\ & + \:{f_{12}}\left( {maximum\:temperature\:lag\:{3_i}} \right) \\ & + \:{f_{13}}\left( {minimum\:temperatur{e_i}} \right) \\ & + \:{f_{14}}\left( {minimum\:temperature\:lag\:{1_i}} \right) \\ & + \:{f_{15}}\left( {minimum\:temperature\:lag\:{2_i}} \right) \\ & + \:{f_{16}}\left( {minimum\:temperature\:lag\:{3_i}} \right) \\ & + \:{f_{17}}\left( {total\:rainfal{l_i}} \right) \\ & + \:{f_{18}}\left( {total\:rainfall\:lag\:{1_i}} \right) \\ & + \:{f_{19}}\left( {total\:rainfall\:lag\:{2_i}} \right) \\ & + \:{f_{20}}\left( {total\:rainfall\:lag\:{3_i}} \right) \\ \end{aligned} $$

(3) Zero-Inflation Model

$$\begin{aligned} \:{\mathrm{log}}\left( {\frac{{{v_i}}}{{1 - {v_i}}}} \right) = \: & \theta {\:_0} + \:{g_1}\left( {latitud{e_i}} \right) \\ & + \:{g_2}\left( {longitud{e_i}} \right) \\ \end{aligned} $$

For district “i”,

$\:\mathrm{log}\left({\mu\:}_{i}\right)$ = log-transformed expected incidence rate.

$\:\mathrm{log}\left({\alpha\:}_{i}\right)$ = log-transformed dispersion parameter of the Gamma distribution.

$\:\mathrm{log}\left(\frac{{v}_{i}}{1-{v}_{i}}\right)$= log-odds of zero incidence due to zero-inflation.

$\:{\beta\:}_{0},{\gamma\:}_{0}$ and $\:{\:\theta\:}_{0}$ are intercept terms. $\:{f}_{1}$ through $\:{f}_{26}$ and $\:{g}_{1}$, $\:{g}_{2}$ represent smooth functions.

The goodness-of-fit of the model was assessed by examining the distribution of the residuals.

### Spatial autocorrelation analysis

To assess spatial dependence in leptospirosis incidence, a Global Moran’s I test was performed using district-level mean incidence rates aggregated over the entire study period. Spatial weights were constructed based on Queen contiguity, in which districts sharing boundaries were considered neighbors. Furthermore, Local Indicators of Spatial Association (LISA) were calculated to identify district-level clustering patterns. District boundary shapefiles were obtained from Humanitarian Data Exchange [21], and spatial joins were conducted to link incidence data to the corresponding polygons.

All data were analyzed using R 4.5.2 software, employing the nasapower, gamlss, mgcv, sp, ggplot2, dplyr, readr gamlss.dist, forcats, sf and spdep packages.

## Results

### Cases incidence and spatial distribution of leptospirosis cases

**Fig. 1:**
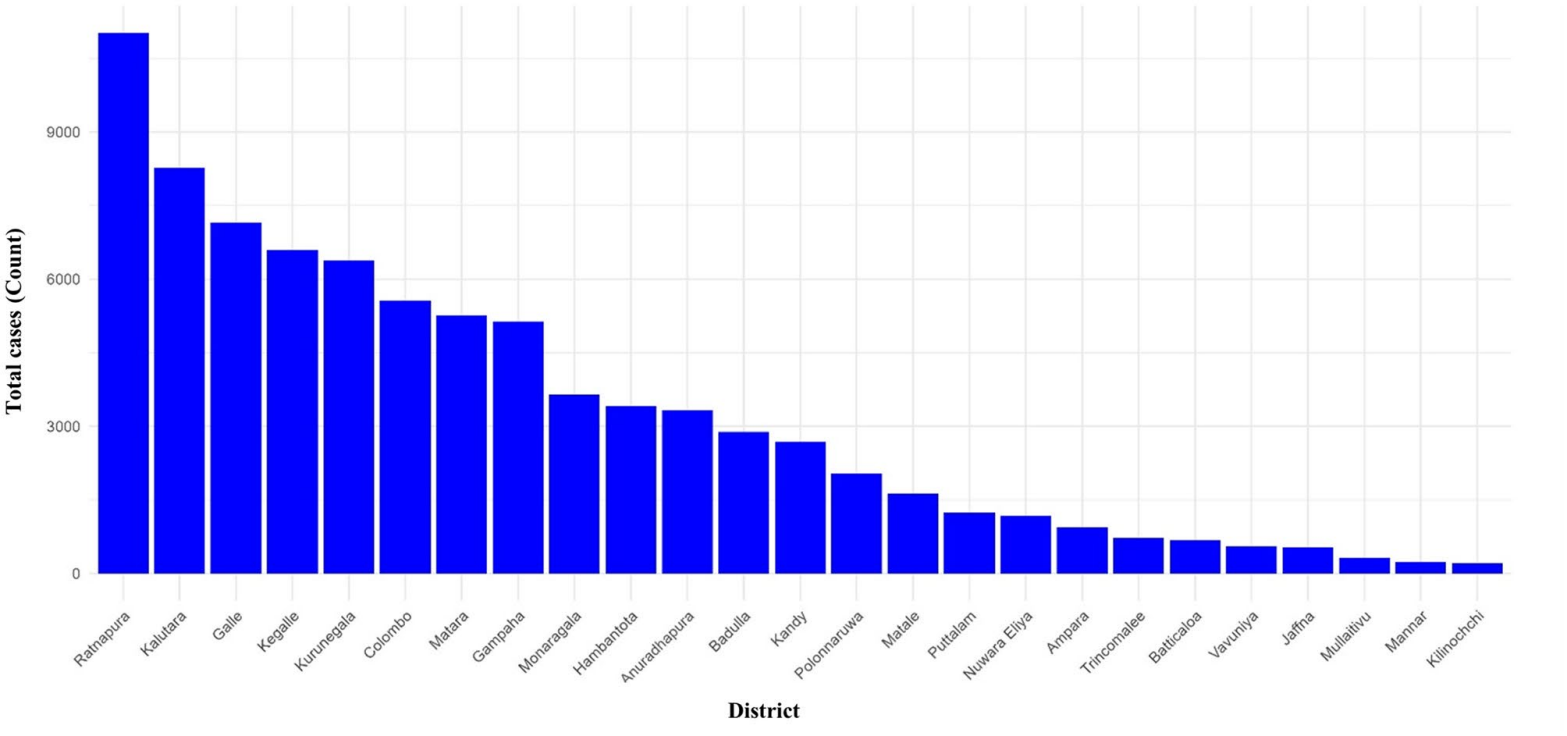
Leptospirosis cases reported in Sri Lanka from 2009 to 2024

**Fig. 2:**
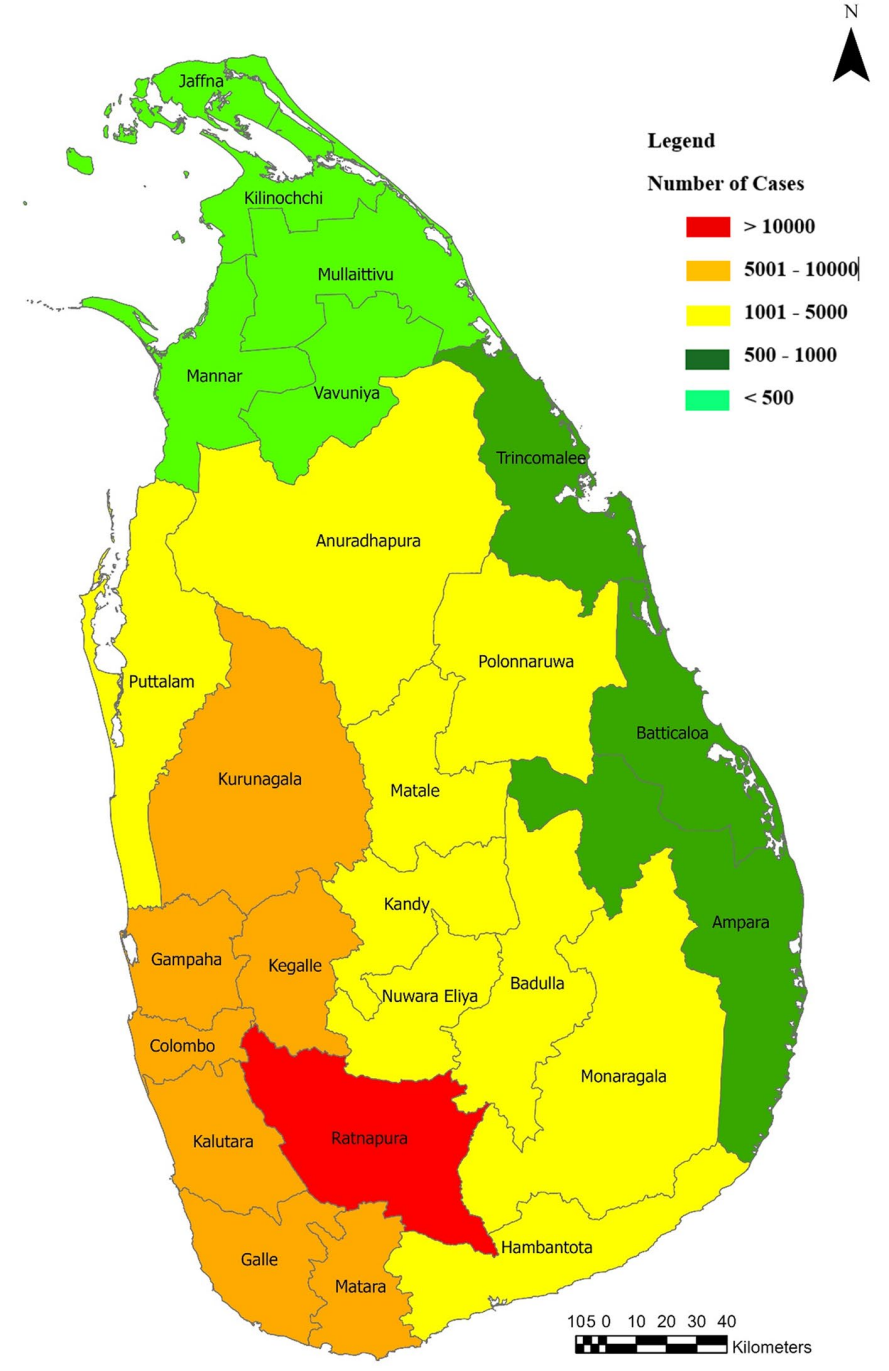
Distribution of leptospirosis cases recorded in different administrative districts in Sri Lanka during 2009–2023

Between 2009 and 2024, a total of 81,629 leptospirosis cases were reported across Sri Lanka. The distribution of cases was markedly heterogeneous across districts. The highest number of cases was reported from Ratnapura District (*n* = 11,019; 13.5% of total cases). Other districts with comparatively high case counts included Kalutara (*n* = 8,261; 10.1%), Galle (*n* = 7,144; 8.7%), Kegalle (*n* = 6,586; 8.1%), and Kurunegala (*n* = 6,382; 7.8%). A lower number of cases was reported from the Northern and Eastern regions of the country, with a gradual increase towards the south-western region (Fig. 1). The highest burden was observed in Ratnapura District, located in the Sabaragamuwa Province. Notably, a clear clustering of high case counts was evident in areas associated with the western slopes of the central hill country (Fig. 2).

### Key predictor effects in the ZAGA-GAMLSS model

The associations between environmental, spatial, and temporal predictors and leptospirosis incidence rates were evaluated using a zero-adjusted gamma (ZAGA) generalized additive model for location, scale, and shape (GAMLSS). The estimated effects for the mean, variability, and zero-inflation components of the model are presented in Tables 1, 2 and 3.

For the expected incidence rate (Table 1), all environmental variables included in the model were statistically significant at least at one lag. Relative humidity at lag 0 was significantly negatively associated with incidence rates (estimate = − 0.0177, *P* < 0.01), whereas relative humidity at lag 1 (estimate = 0.0448, *P* < 0.001) and lag 2 (estimate = 0.0219, *P* < 0.01) showed significant positive associations. The mean temperature at lag 3 was significantly positively associated with incidence rates (estimate = 0.346, *P* < 0.05). Maximum temperature was significantly negatively associated with incidence rates at lag 0 (estimate = − 0.188, *P* < 0.01) and lag 3 (estimate = − 0.153, *P* < 0.05), while minimum temperature at lag 3 also showed a significant negative association (estimate = − 0.161, *P* < 0.05).

Total rainfall exhibited significant positive associations with incidence rates at lag 1 (estimate = 0.00045, *P* < 0.001) and lag 3 (estimate = 0.00027, *P* < 0.05). In addition, both latitude (estimate = 0.0761, *P* < 0.001) and longitude (estimate = 0.0869, *P* < 0.001) were significantly positively associated with incidence rates. Temporal autocorrelation was observed, with incidence rates at lag 1 (estimate = 0.15, *P* < 0.001) and lag 12 (estimate = 0.0236, *P* < 0.001) showing significant positive associations with current incidence.

For the scale parameter, which represents the dispersion of incidence rates (Table 2), relative humidity at a three‑month lag was the only predictor that showed a statistically significant association, with a negative effect (estimate = − 0.01977, *P* < 0.01).

For the zero-inflation component of the model (Table 3), latitude (estimate = 1.20275, *P* < 0.001) and longitude (estimate = 0.70892, *P* < 0.001) were significantly positively associated with the probability of excess zeros in the incidence data.

**Table 1:** Parameter and risk estimates of the expected incidence rate model

Variable	Estimate	Std. Error	Risk Estimate	95% confidence interval of the risk estimate	t value	*P* value	Significance Level
(Intercept)	-103.00000	5.21000	-	-	-19.755	< 2e-16	***
pb(mean relative humidity)	-0.01770	0.00659	0.9825	0.9699–0.9953	-2.679	0.00742	**
pb(mean relative humidity lag1)	0.04480	0.00823	1.0459	1.0291–1.0629	5.448	5.36E-08	***
pb(mean relative humidity lag2)	0.02190	0.00835	1.0221	1.0055–1.0390	2.619	0.008839	**
pb(mean relative humidity lag3)	0.00066	0.00677	1.0007	0.9875–1.0140	0.097	0.922403	
pb(mean temperature)	0.23300	0.15000	1.2618	0.9413–1.6913	1.555	0.119943	
pb(mean temperature lag1)	0.17900	0.18300	1.1965	0.8357–1.7131	0.979	0.327442	
pb(mean temperature lag2)	-0.14800	0.19300	0.8621	0.5902–1.2592	-0.768	0.442593	
pb(mean temperature lag3)	0.34600	0.15600	1.4127	1.0405–1.9180	2.215	0.026837	*
pb(maximum temperature)	-0.18800	0.06930	0.8284	0.7233–0.9489	-2.717	0.006611	**
pb(maximum temperature lag1)	-0.02250	0.08440	0.9778	0.8286–1.1538	-0.266	0.790204	
pb(maximum temperature lag2)	0.11400	0.08910	1.1206	0.9411–1.3344	1.278	0.201247	
pb(maximum temperature lag3)	-0.15300	0.07360	0.8582	0.7429–0.9914	-2.077	0.037816	*
pb(minimum temperature)	-0.15000	0.08110	0.861	0.7345–1.0092	-1.847	0.064852	
pb(minimum temperature lag1)	-0.03730	0.09490	0.9633	0.7999–1.1602	-0.394	0.693817	
pb(minimum temperature lag2)	0.07540	0.10100	1.0783	0.8850–1.3139	0.749	0.454066	
pb(minimum temperature lag3)	-0.16100	0.08090	0.8516	0.7268–0.9979	-1.986	0.047041	*
pb(total rainfall)	0.00005	0.00011	1	0.9998–1.0003	0.429	0.668258	
pb(total rainfall lag1)	0.00045	0.00012	1.0004	1.0002–1.0007	3.782	0.000158	***
pb(total rainfall lag2)	0.00007	0.00012	1.0001	0.9998–1.0003	0.621	0.534796	
pb(total rainfall lag3)	0.00027	0.00011	1.0003	1.0000–1.0005	2.382	0.017274	*
pb(Year)	0.04410	0.00253	1.0451	1.0399–1.0503	17.41	< 2e-16	***
pb(Month)	0.02010	0.00461	1.0203	1.0111–1.0295	4.355	1.36E-05	***
pb(Latitude)	0.07610	0.01270	1.0791	1.0526–1.1063	6.006	2.06E-09	***
pb(Longitude)	0.08690	0.02070	1.0908	1.0473–1.1360	4.188	2.87E-05	***
pb(Incidence rate lag1)	0.15000	0.00388	1.1615	1.1527–1.1703	38.637	< 2e-16	***
pb(Incidence rate lag12)	0.02360	0.00452	1.0239	1.0149–1.0330	5.231	1.77E-07	***

Significance codes: *** *p* < 0.001; ** *p* < 0.01; * *p* < 0.05

**Table 2:** Parameter and risk estimates of the dispersion model

Variable	Estimate	Std. Error	Risk Estimate	95% confidence interval of the risk estimate	t value	*P* value	Significance Level
(Intercept)	3.626	0.862			4.206	2.65E-05	***
pb(mean relative humidity)	-0.009036	0.006913	0.991005	0.9777–1.0045	-1.307	0.19126	
pb(mean relative humidity lag1)	0.0116	0.00888	1.011668	0.9942–1.0294	1.306	0.19157	
pb(mean relative humidity lag2)	-0.01276	0.008894	0.987321	0.9703–1.0047	-1.435	0.15129	
pb(mean relative humidity lag3)	-0.01977	0.007085	0.980424	0.9669–0.9941	-2.79	0.00529	**
pb(mean temperature)	0.1707	0.1605	1.186135	0.866–1.6246	1.064	0.28755	
pb(mean temperature lag1)	-0.1277	0.1997	0.880117	0.595–1.3018	-0.64	0.52249	
pb(mean temperature lag2)	-0.2677	0.1999	0.765137	0.5171–1.1321	-1.339	0.1806	
pb(mean temperature lag3)	0.1607	0.1669	1.174333	0.8467–1.6288	0.963	0.33577	
pb(maximum temperature)	-0.05922	0.07446	0.942499	0.8145–1.0906	-0.795	0.42647	
pb(maximum temperature lag1)	0.03504	0.09139	1.035661	0.8658–1.2388	0.383	0.7014	
pb(maximum temperature lag2)	0.1066	0.09164	1.112489	0.9296–1.3314	1.163	0.24489	
pb(maximum temperature lag3)	-0.09787	0.07888	0.906767	0.7769–1.0584	-1.241	0.21476	
pb(minimum temperature)	-0.09598	0.08698	0.908482	0.7661–1.0773	-1.103	0.26987	
pb(minimum temperature lag1)	0.1184	0.1044	1.125694	0.9174–1.3813	1.134	0.25701	
pb(minimum temperature lag2)	0.07593	0.1047	1.078887	0.8787–1.3246	0.725	0.46837	
pb(minimum temperature lag3)	-0.07874	0.08647	0.92428	0.7802–1.095	-0.911	0.36255	
pb(total rainfall)	-0.00002204	0.0001223	0.999978	0.9997–1.0002	-0.18	0.85698	
pb(total rainfall lag1)	0.0001308	0.0001263	1.000131	0.9999–1.0004	1.035	0.3006	
pb(total rainfall lag2)	0.0000145	0.0001258	1.000015	0.9998–1.0003	0.115	0.90827	
pb(total rainfall lag3)	0.00001991	0.0001166	1.00002	0.9998–1.0002	0.171	0.86443	

Significance codes: *** *p* < 0.001; ** *p* < 0.01; * *p* < 0.05

**Table 3:** Parameter and risk estimates of the zero-inflation model

Variable	Estimate	Std. Error	Risk Estimate	95% confidence interval of the risk estimate	t value	*P* value	Significance Level
(Intercept)	-68.6886	7.72304			-8.894	< 2e-16	***
pb(Latitude)	1.20275	0.056	0.769014	0.7489–0.7879	21.477	< 2e-16	***
pb(Longitude)	0.70892	0.09294	0.670162	0.6287–0.7091	7.628	2.91E-14	***

Significance codes: *** *p* < 0.001; ** *p* < 0.01; * *p* < 0.05

### Goodness-of-fit of the model

**Fig. 3:**
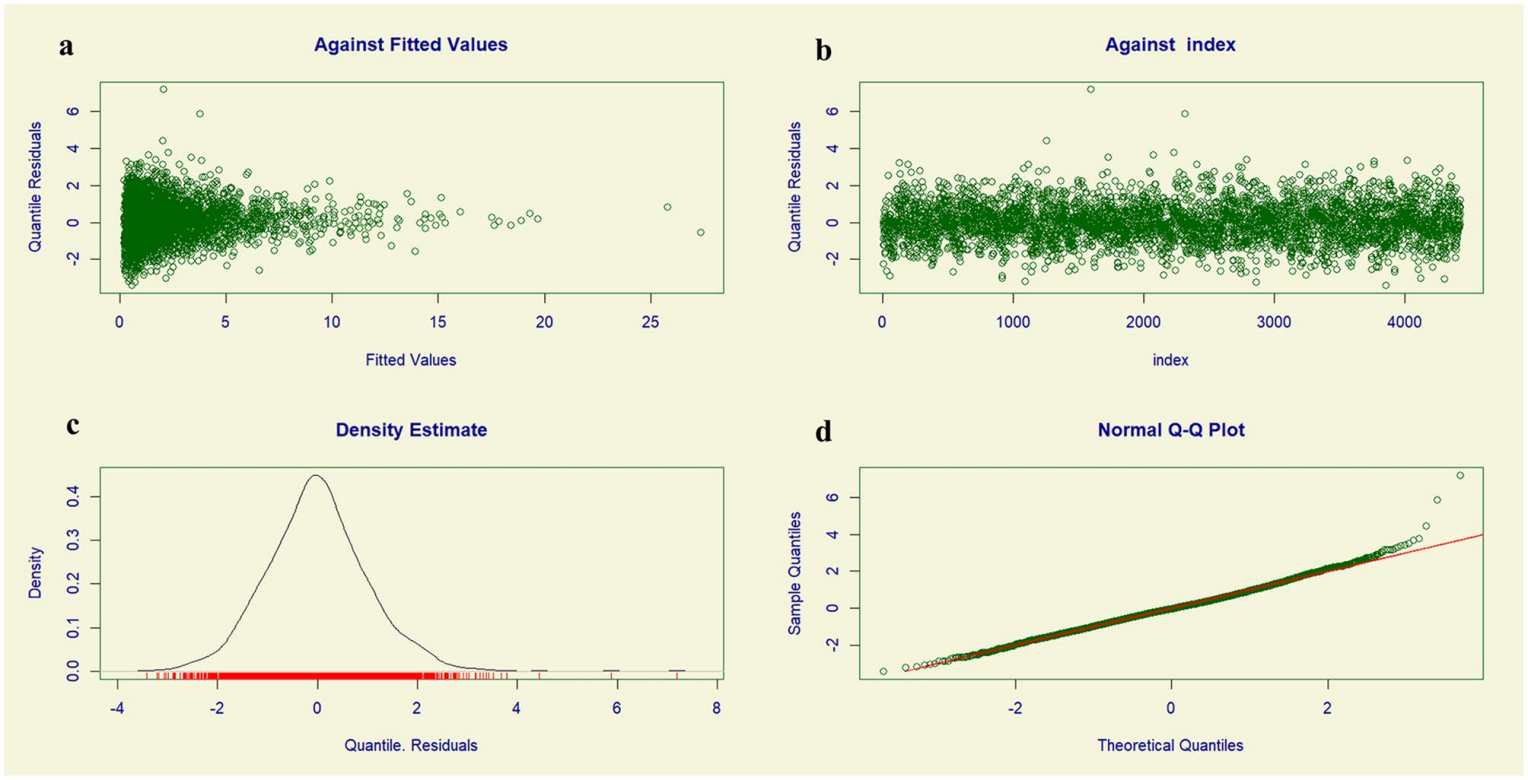
Residual diagnostic plots for evaluating the adequacy of the Zero-Adjusted Gamma (ZAGA) Generalized Additive Model for Location, Scale, and Shape (GAMLSS): **(a)** Residuals vs. fitted values; **(b)** Residuals vs. observation index; **(c)** Density plot of residuals; and **(d)** Normal Q-Q plot

Residual diagnostic plots indicated that the Zero-Adjusted Gamma (ZAGA) GAMLSS model provided a satisfactory fit to the data. As shown in Fig. 3a, the residuals were randomly scattered around zero when plotted against fitted values, suggesting no major concerns with non-linearity or heteroscedasticity. Figure 3b, which displays residuals against the observation index, revealed no discernible patterns, indicating an absence of significant autocorrelation. The density plot of residuals (Fig. 3c) approximated a normal distribution, forming a bell-shaped curve. Similarly, the normal Q–Q plot (Fig. 3d) showed that residuals closely followed the 45-degree reference line, supporting the assumption of normality. Collectively, these diagnostic plots did not reveal any critical violations of model assumptions, providing evidence that the ZAGA GAMLSS model adequately captured the underlying structure of the data.

### Temporal distribution of leptospirosis incidence

**Fig. 4:**
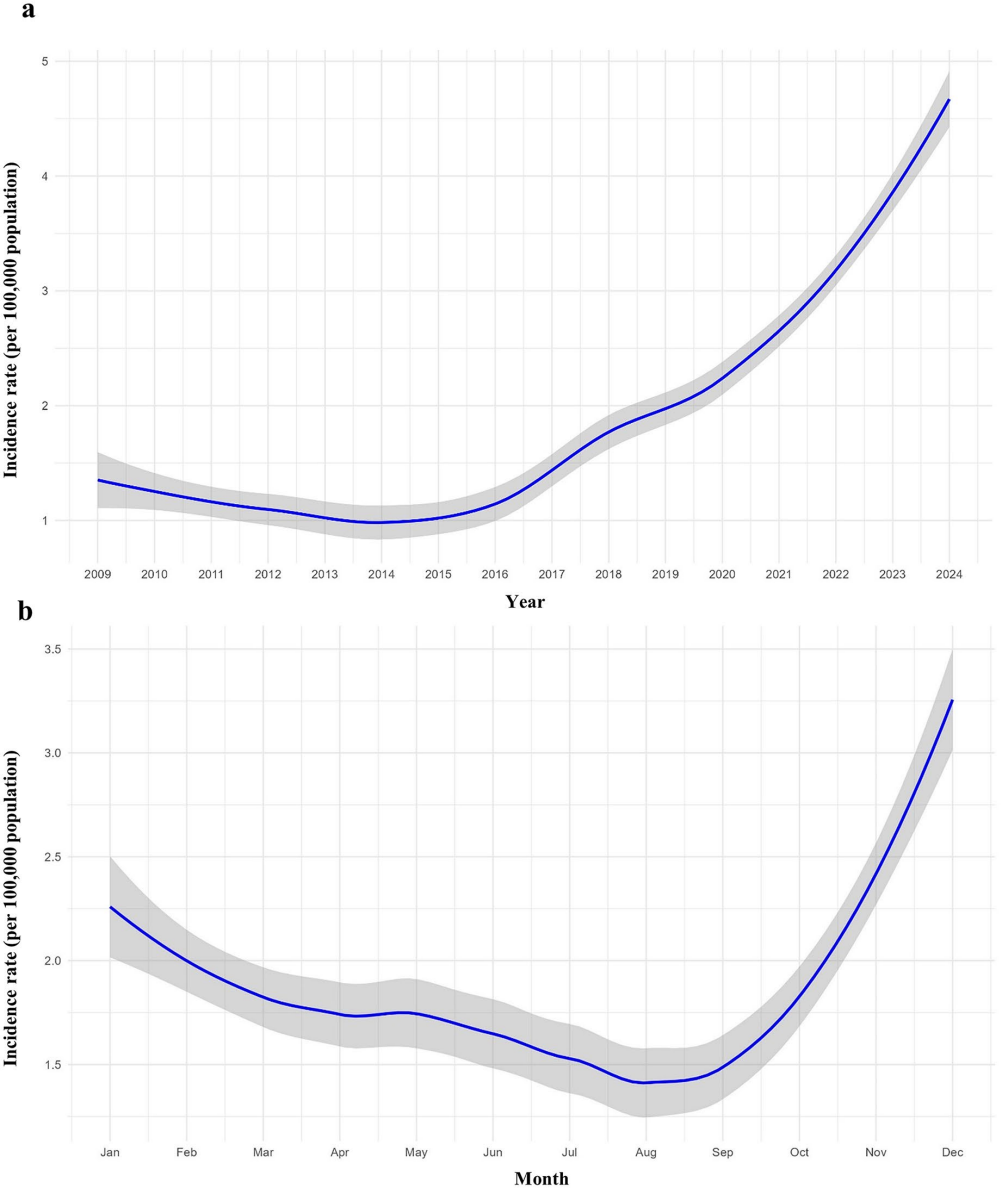
Trend-lines of leptospirosis incidence rates per 100,000 population in Sri Lanka, with shaded areas representing the 95% confidence intervals: **(a)** annual trends and **(b**) monthly trends

Leptospirosis incidence exhibited significant annual (estimate = 0.0441, *P* < 0.001) and seasonal variation (estimate = 0.0201, *P* < 0.001) (Table 1). The annual trend of incidence rates per 100,000 population from 2009 to 2024 is presented in Fig. 4a. A marked and steep increase in incidence was observed beginning in the 2015–2016 period, with the upward trend continuing through 2024.

The monthly trend (Fig. 4b) showed that incidence rates typically peaked towards the end of each year. From September onwards, a rapid rise in incidence was consistently observed, indicating a pronounced seasonal pattern.

### Spatial autocorrelation analysis

A Global Moran’s I test indicated significant positive spatial autocorrelation in district-level mean leptospirosis incidence rates (Moran’s I = 0.332, standard deviate = 3.07, *p* = 0.0011), suggesting that districts with similar incidence rates tended to cluster geographically.

**Fig. 5:**
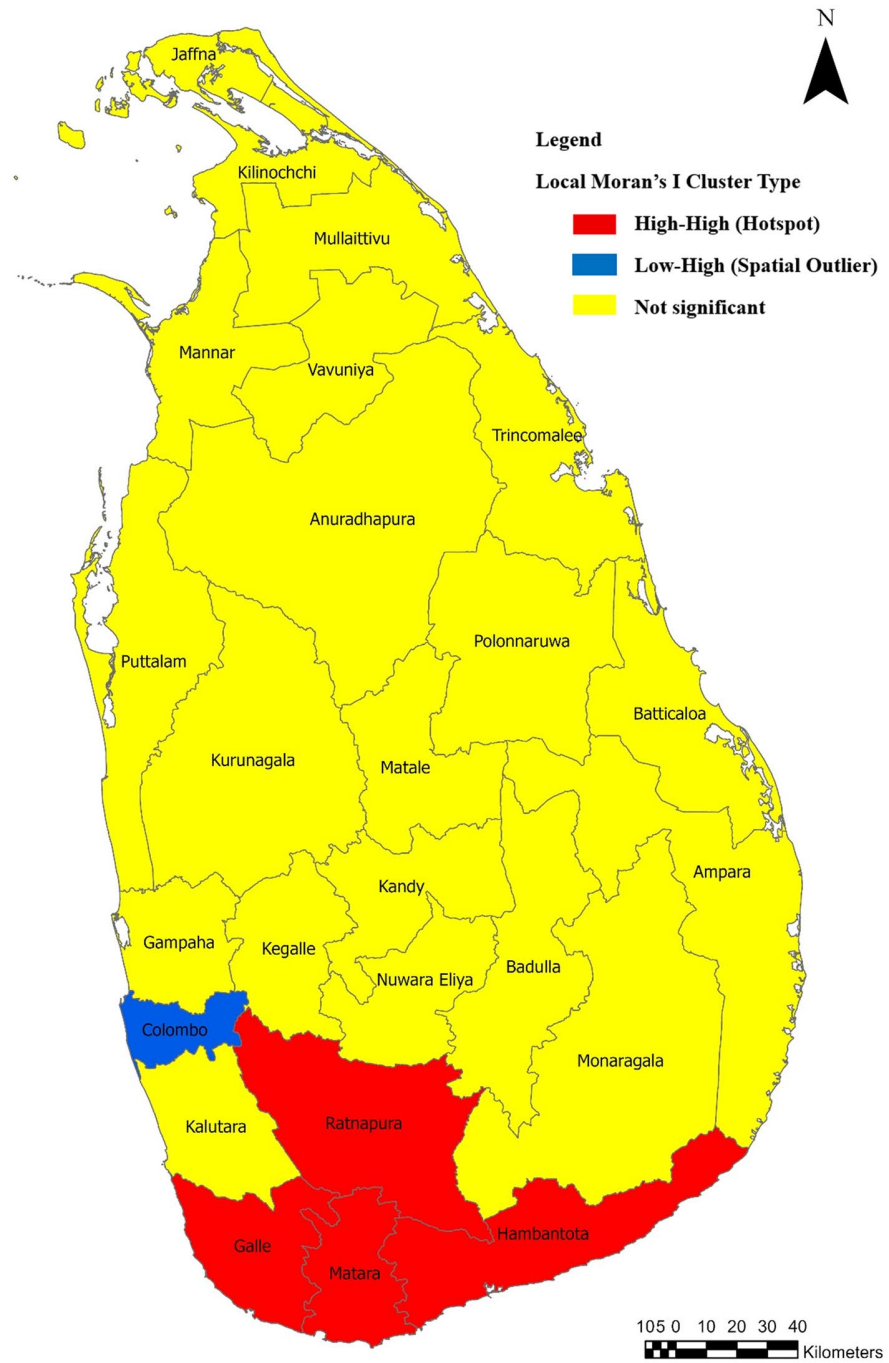
Spatial distribution of leptospirosis incidence clusters across districts in Sri Lanka based on Local Moran’s I (LISA) analysis (https://data.humdata.org/dataset/sri-lanka-administrative-levels-0-4-boundaries)

Local Moran’s I (LISA) analysis identified district-level clustering patterns. Four districts, Ratnapura, Galle, Matara, and Hambantota, were classified as high-high clusters (hotspots), indicating higher-than-average incidence surrounded by neighbouring districts with similarly high incidence rates. Colombo was identified as a low-high spatial outlier, reflecting a lower-than-average incidence relative to its neighbours with high incidence. The remaining districts showed no statistically significant local autocorrelation. The spatial distribution of these clusters is illustrated in Fig. 5.

### District-wise trends in leptospirosis incidence rates

**Fig. 6:**
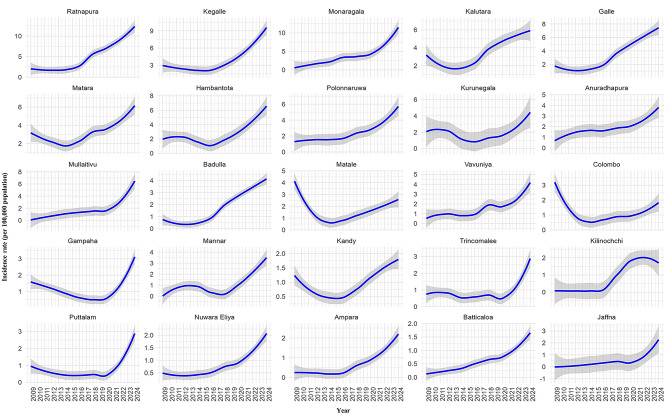
Annual trends in Leptospirosis incidence rates (per 100,000 population) by district, Sri Lanka, 2009–2023, with shaded areas representing 95% confidence intervals

Analysis of annual incidence rates from 2009 to 2024 revealed considerable heterogeneity in leptospirosis incidence across districts (Fig. 6). Ratnapura, Kegalle, and Monaragala recorded the highest incidence rates, each demonstrating an overall increasing trend over the study period. Although the magnitude of incidence varied between districts, most showed a generally increasing pattern. A few districts exhibited distinct temporal trajectories. Matale and Colombo experienced a pronounced decline from 2009 to 2014, followed by a gradual increase thereafter. In Kilinochchi, incidence rose gradually between 2015 and 2020 and then declined modestly after 2020.

**Fig. 7:**
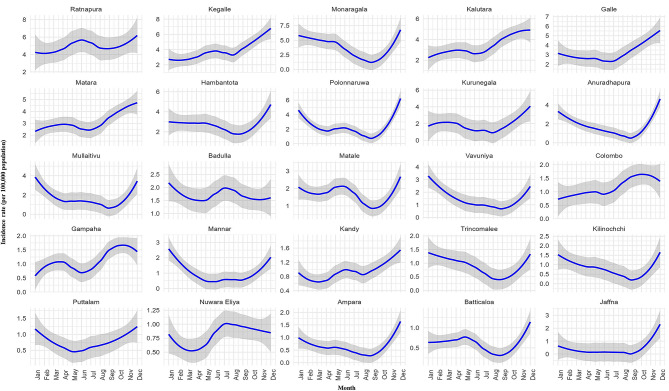
Monthly trends in Leptospirosis incidence rates (per 100,000 population) by district, Sri Lanka, 2009–2023, with shaded areas denoting 95% confidence intervals

When examining the monthly trends across districts (Fig. 7), several distinct temporal patterns were observed, differing from the annual trends. Ratnapura, Badulla, and Kandy exhibited a similar pattern characterized by a sharp increase in incidence around the middle of the year, followed by a subsequent decline. In contrast, Matara and Gampaha showed an opposite trend, with a midyear decrease followed by an increase toward the end of the year. Monaragala, Hambantota, Polonnaruwa, Anuradhapura, Mullaitivu, Matale, Trincomalee, Kilinochchi, Ampara, Batticaloa, and Jaffna displayed comparable patterns, showing a gradual decline in incidence from January to around August–September, followed by a marked rise toward the end of the year. Most of the remaining districts demonstrated a gradual increase throughout the year with minor fluctuations. However, Nuwara Eliya exhibited a distinct pattern, with a decline in incidence from January to April, a rapid rise peaking around July–August, and a subsequent decreasing trend toward the end of the year.

## Discussion

This study provides the first comprehensive national-level synthesis of leptospirosis epidemiology in Sri Lanka over a 16-year period (2009–2024), integrating temporal, spatial, and climatic perspectives. It reveals pronounced spatial and temporal variability in leptospirosis incidence across Sri Lanka, highlighting the critical role of climatic drivers in transmission dynamics of leptospirosis. The highest burden observed in the south-western part of the country is consistent with previous national studies reporting elevated incidence within the wet zone, where high rainfall and humidity predominate [22]. Similar spatial patterns have also been described in other tropical countries with monsoonal climates, such as Thailand and the Philippines, where wet agricultural regions emerge as persistent hotspots [23–25]. The clustering identified along the western slopes of the central hill country aligns with earlier evidence highlighting the contribution of watershed networks, plantation agriculture, and paddy cultivation in facilitating human exposure to contaminated water [26].

The modeling results revealed several important lagged relationships, with each lag in this study corresponding to a one-month interval. These lagged effects offer insight into the temporal sequence linking climate exposure, environmental contamination, and human infection. Relative humidity showed an immediate negative association at lag 0, but positive associations at lags 1 and 2. The negative short-term effect may reflect reduced exposure during and immediately after heavy rainfall when agricultural labor is halted or access to fields is limited. However, the positive effects at one and two months correspond with sustained moist conditions that favor the survival of *Leptospira* in surface water and soil. This finding is consistent with earlier laboratory and environmental studies, which demonstrate that *Leptospira* persist for several weeks in humid environments [27–29], and with ecological analyses from Brazil and India, showing delayed increases in incidence one to two months after high-humidity periods [30, 31].

Temperature displayed nuanced lagged associations. The positive effect of mean temperature at lag 3 suggests that moderate temperatures three months prior support bacterial persistence and subsequent transmission, aligning with studies indicating optimal survival in temperate, non-extreme conditions [32]. The negative associations of maximum and minimum temperatures, also at lag 3, suggest that temperature extremes, whether hot or cold, three months prior, may reduce environmental viability. These monthly lag effects support the hypothesis that temperature influences transmission indirectly by shaping the environmental reservoir over preceding months, rather than driving immediate infection.

Rainfall exhibited significant positive associations at lags 1 and 3. The one-month lag likely reflects contamination of water sources following rainfall events, with exposure occurring once agricultural activity resumes. The three-month lag may correspond to persistent standing water, prolonged saturation of paddy fields, and delayed human contact with contaminated reservoirs. Previous Sri Lankan research, which linked monsoonal flooding to an increased incidence within one to two months [22, 33, 34], and international studies reporting similar monthly lag structures [35–37], align with these results.

The temporal structure of leptospirosis incidence was further reflected in the significant annual and seasonal effects. Peaks were consistent with south-western monsoon and inter-monsoon periods, corroborating earlier national surveillance findings [22, 33, 38]. The autoregressive effects at lags 1 and 12, representing one-month and one-year recurrence, demonstrate monthly continuity in transmission and a recurring seasonal cycle. The one-month autoregression may be related to ongoing infections in occupational groups, while the twelve-month repetition highlights monsoon-driven seasonality.

The spatial components of the model indicated that latitude and longitude were significantly and positively associated with both the incidence rate parameters and the zero-inflation component. This suggests that spatial heterogeneity plays a critical role in disease distribution, where geographical gradients likely reflect underlying environmental, land-use, and population-level differences that influence exposure risk. The presence of significant spatial autocorrelation was further supported by Moran’s I and LISA analyses, which identified high–high (hotspot) clusters in Ratnapura, Galle, Matara, and Hambantota. The low–high (spatial outlier) pattern observed in Colombo highlights comparatively lower incidence within urban centers, despite their proximity to rural high-risk regions. This contrast may reflect reduced agricultural exposure, improved sanitation, and enhanced public health infrastructure in urban settings.

Collectively, the results demonstrate that climatic conditions influence leptospirosis transmission through mechanisms operating over one to three-month periods: rainfall initiates environmental contamination, humidity sustains leptospiral viability, and moderate temperatures support persistence. These lag structures emphasize that meteorological events do not produce immediate incidence surges; rather, they create conditions that elevate risk in subsequent months. When combined with agricultural cycles and monsoon patterns, these monthly delays produce predictable seasonal peaks and persistent spatial hotspots. Such insights reinforce the value of early-warning systems based on monthly rainfall and humidity forecasts and support geographically and seasonally targeted interventions in high-risk regions.

In this study, a Zero-Adjusted Gamma (ZAGA) distribution within the GAMLSS framework was selected to model leptospirosis incidence because the data exhibited substantial zero inflation (15% of months with zero reported cases) and strong non-linear relationships with climatic predictors. Although correlation-based analyses can characterize simple associations, they do not accommodate zero inflation or the heteroskedasticity present in these data. Likewise, generalized linear mixed models (GLMMs) are appropriate for hierarchical structures but typically rely on Poisson or negative binomial distributions that assume count data and were not well suited to the continuous incidence rates and excess zeros observed here. More explicitly predictive approaches, such as machine learning methods, can capture complex patterns but often do so at the cost of interpretability and formal parameter estimation. Because our primary objective was to quantify and interpret the effects of climatic variables rather than to maximize predictive performance, the ZAGA–GAMLSS specification provided an appropriate solution. This framework enabled joint modeling of the mean, dispersion, and zero-inflation components, yielding a flexible and interpretable approach for assessing climatic influences and geographic heterogeneity in leptospirosis transmission.

This study has notable strengths, including the use of 16 years of nationally representative surveillance data and the application of robust spatiotemporal and climate modeling approaches. Nevertheless, limitations should be considered. This study was based on national surveillance data published by the Epidemiology Unit of the Ministry of Health, Sri Lanka. It is clearly indicated that the disease notification is done on clinical suspicion following a defined criterion [18]. Further, the laboratory tests will be conducted by the Medical Research institute, Sri Lanka whenever there is a doubt in the clinical suspicion based on the request made by the clinician at the reporting site [19]. Therefore, laboratory results for all notified cases are not available in the national database which could be acknowledged as a limitation in this study. However, it is assured that there is no duplication of data with laboratory confirmation. Underreporting of leptospirosis is well recognized globally, and the extent of under-ascertainment is likely to vary by geographic region, healthcare access, clinician awareness, and laboratory diagnostic capacity. Furthermore, temporal and regional variations in laboratory confirmation practices may influence observed trends in reported cases.

Beyond these surveillance constraints, diagnostic limitations must also be acknowledged. Laboratory confirmation in Sri Lanka primarily relies on the Microscopic Agglutination Test (MAT), performed at the Medical Research Institute (MRI), using 12 locally prevalent serovars. However, this information is not publicly available. Therefore, MAT results were not accessible in this study. In addition, IgM ELISA, although widely used as a rapid diagnostic tool, does not always indicate an acute infection, as IgM antibodies can persist for weeks to months. Cases confirmed solely by IgM ELISA may therefore represent past exposure, potentially biasing spatiotemporal analyses. The PCR and culture offer greater specificity but are less widely available, meaning most confirmations rely on serology. These diagnostic limitations should be considered when interpreting our findings, and future studies should incorporate PCR where feasible, while advocating for greater transparency in the composition of MAT panels.

From a public health perspective, several implications emerge. First, incorporating meteorological data into routine surveillance could strengthen early warning systems and predictive modeling for leptospirosis outbreaks. Second, geographically tailored prevention strategies are warranted, with high-risk districts prioritized for interventions such as rodent control programs, personal protective equipment for agricultural workers, and targeted health education campaigns. Third, findings reinforce the importance of embedding leptospirosis prevention within national climate adaptation strategies, acknowledging the demonstrated links between climate variability and disease transmission.

Although the present study focused on human surveillance data and climatic variables, leptospirosis is a quintessential One Health disease, shaped by complex interactions among humans, animal reservoirs, and the environment. Future research in Sri Lanka should therefore adopt an integrated One Health framework that incorporates data on animal reservoirs, including rodents, livestock, and domestic dogs, alongside agricultural and land-use characteristics such as paddy cultivation practices and the flooding of agricultural areas. Additionally, environmental and remote-sensing indicators, such as flood extent and land-cover patterns, should be systematically integrated. To coherently incorporate and prioritize these heterogeneous data streams, multi-criteria decision analysis frameworks such as the GIZ method and Delphi–Entropy approaches could be employed, as demonstrated in previous vector-borne disease studies within the Sri Lankan context [39–41]. These integrative approaches would complement explanatory epidemiological models by providing transparent and structured tools for risk stratification, surveillance planning, and intervention prioritization. Framing leptospirosis within this broader One Health perspective not only enhances conceptual depth but also offers a pragmatic roadmap for strengthening future surveillance systems, research agendas, and disease control programmes in Sri Lanka.

## Conclusion

This study presents the first comprehensive national synthesis of leptospirosis in Sri Lanka, revealing that the disease has not only persisted but also evolved in terms of intensity and geographic distribution over the past 16 years, with hotspots identified in the districts of Ratnapura, Galle, Matara, and Hambantota. The strong and consistent associations with climatic parameters highlight leptospirosis as one of the clearest climate-sensitive health threats in the country. Strong positive associations were detected with high relative humidity at one and two months lag periods. Similarly, high total rainfall was positively associated with the incidence rate at one and three-month lag periods. In contrast, negative associations are observed at a three-month lag with temperature extremes. These findings call for urgent integration of meteorological data into surveillance, predictive modeling, and preparedness planning. Without climate-adaptive interventions and district-targeted control measures, Sri Lanka is likely to face an escalating burden of leptospirosis under changing climate conditions. The evidence presented here should serve as a critical warning for policymakers to act now to prevent the widening impact of this neglected tropical disease.

## Supplementary Information

Below is the link to the electronic supplementary material.

Supplementary Material 1: 1- Model Comparison

## Data Availability

The datasets supporting the conclusion of this article are included within the article.

## Abbreviations

NASA POWER: National Aeronautics and Space Administration–Prediction Of Worldwide Energy Resources
ZAGA: Zero–Adjusted Gamma
GAMLSS: Generalized Additive Model for Location, Scale, and Shape
GLMM: Generalized Linear Mixed Model
AIC: Akaike Information Criterion
BIC: Bayesian Information Criterion
Q-Q: Quantile-Quantile
LISA: Local Indicators of Spatial Association
WHO: World Health Organization
MAT: Microscopic Agglutination Test
ELISA: Enzyme–Linked Immunosorbent Assay
PCR: Polymerase Chain Reaction
IgM: Immunoglobulin M
MRI: Medical Research Institute

## References

[1] Pinto GV, Senthilkumar K, Rai P, Kabekkodu SP, Karunasagar I, Kumar BK. Current methods for the diagnosis of leptospirosis: issues and challenges. J Microbiol Methods. 2022;195:106438. 10.1016/J.MIMET.2022.106438. 35248601 10.1016/j.mimet.2022.106438

[2] Pal M, Roba Bulcha M, Bune WM, Bulcha MR. Leptospirosis and One Health Perspective. Am J Public Health Res. 2021;9(3):180–3. 10.12691/AJPHR-9-4-9.

[3] Vijayachari P, Sugunan AP, Shriram AN. Leptospirosis: an emerging global public health problem. J Biosci. 2008;33:557–69. 10.1007/S12038-008-0074-Z/METRICS. 19208981 10.1007/s12038-008-0074-z

[4] WHO. Human Leptospirosis: Guidance for Diagnosis, Surveillance and Control. 2003. Accessed on 11 October 2025.

[5] Warnasekara JN, Agampodi S. Leptospirosis in Sri Lanka. Sri Lankan J Infect Dis. 2017;7:67. 10.4038/SLJID.V7I2.8155.

[6] Agampodi SB, Peacock SJ, Thevanesam V, Nugegoda DB, Smythe L, Thaipadungpanit J, et al. Leptospirosis outbreak in Sri Lanka in 2008: lessons for assessing the global burden of disease. Am J Trop Med Hyg. 2011;85:471–8. 10.4269/AJTMH.2011.11-0276. 21896807 10.4269/ajtmh.2011.11-0276 PMC3163869

[7] Temporal & Spatial Trends of Leptospirosis cases in Sri Lanka. 2017. 10.24940/ijird/2017/v6/i6/116041-268418-1-SM.

[8] Lau CL, Smythe LD, Craig SB, Weinstein P. Climate change, flooding, urbanisation and leptospirosis: fuelling the fire? Trans R Soc Trop Med Hyg. 2010;104:631–8. 10.1016/J.TRSTMH.2010.07.002. 20813388 10.1016/j.trstmh.2010.07.002

[9] Torgerson PR, Hagan JE, Costa F, Calcagno J, Kane M, Martinez-Silveira MS, et al. Global burden of leptospirosis: estimated in terms of disability adjusted life years. PLoS Negl Trop Dis. 2015;9:e0004122. 10.1371/JOURNAL.PNTD.0004122. 26431366 10.1371/journal.pntd.0004122 PMC4591975

[10] Epidemiology Unit. https://www.epid.gov.lk/leptospirosis. Accessed 5 Oct 2025.

[11] Warnasekara J, Koralegedara I, Agampodi S. Estimating the burden of leptospirosis in Sri Lanka; A systematic review. BMC Infect Dis. 2019;19:1–12. 10.1186/S12879-018-3655-Y/TABLES/5. 30727968 10.1186/s12879-018-3655-y PMC6364467

[12] Ekanayake WEMDT, Nawarathna LS. A model for predicting confirmed leptospirosis cases in Sri Lanka. J Sci Univ Kelaniya. 2024;17:73–91. 10.4038/josuk.v17i2.8109.

[13] Geekiyanage N, Vithanage M. State of the environment, environmental challenges and governance in Sri Lanka. 2015. 10.4324/9781315717081-15.

[14] Punyawardena BVR. Climate. 2020;13:13–22. 10.1007/978-3-030-44144-9_2.

[15] Edirisinghe EANV, Pitawala HMTGA, Dharmagunawardhane HA, Wijayawardane RL. Spatial and Temporal variation in the stable isotope composition (δ18O and δ2H) of rain across the tropical Island of Sri Lanka. Isot Environ Health Stud. 2017;53:628–45. 10.1080/10256016.2017.1304936;ISSUE:ISSUE:DOI. 10.1080/10256016.2017.1304936 28385072

[16] Epidemiology Unit. https://www.epid.gov.lk/weekly-epidemiological-report. Accessed 25 Dec 2025.

[17] Sparks AH, Nasapower. A NASA POWER Global Meteorology, Surface Solar Energy and Climatology Data Client for R. 2018. 10.21105/joss.01035.

[18] National Guidelines on Management of Leptospirosis. Epidemiology Unit, Ministry of Health, Nutrition and Indigenous Medicine, Sri Lanka; 2016.

[19] Epidemiology Unit, Ministry of Health and Indigenous Medicine (M of HN& IM). Weekly Epidemiological Report Vol. 49 No. 22. Colombo: Ministry of Health and Indigenous Medicine; 2022.

[20] Stasinopoulos DM, Rigby RA. Generalized additive models for location scale and shape (GAMLSS) in R. J Stat Softw. 2008;23:1–46. 10.18637/JSS.V023.I07.

[21] Sri Lanka - Subnational Administrative Boundaries | Humanitarian Dataset | HDX. https://data.humdata.org/dataset/cod-ab-lka. Accessed 21 Dec 2025.

[22] Warnasekara J, Agampodi S, Rupika Abeynayake R. Time series models for prediction of leptospirosis in different climate zones in Sri Lanka. PLoS ONE. 2021;16:e0248032. 10.1371/JOURNAL.PONE.0248032. 33989292 10.1371/journal.pone.0248032 PMC8121312

[23] Mohd Radi MF, Hashim JH, Jaafar MH, Hod R, Ahmad N, Nawi AM, et al. Leptospirosis outbreak after the 2014 major flooding event in Kelantan, malaysia: A Spatial-Temporal analysis. Am J Trop Med Hyg. 2018;98:1281–95. 10.4269/AJTMH.16-0922. 29532771 10.4269/ajtmh.16-0922 PMC5953347

[24] Chadsuthi S, Chalvet-Monfray K, Geawduanglek S, Wongnak P, Cappelle J. Spatial–temporal patterns and risk factors for human leptospirosis in Thailand, 2012–2018. Sci Rep. 2022;12(1):5066. 10.1038/s41598-022-09079-y. 10.1038/s41598-022-09079-y PMC8948194 35332199

[25] Luenam A, Puttanapong N. Spatial and statistical analysis of leptospirosis in Thailand from 2013 to 2015. Geospat Health. 2019;14:121–7. 10.4081/GH.2019.739. 10.4081/gh.2019.739 31099522

[26] Schønning MH, Phelps MD, Warnasekara J, Agampodi SB, Furu P. A case–control study of environmental and occupational risks of leptospirosis in Sri Lanka. EcoHealth. 2019;16(3):534–43. 10.1007/S10393-019-01448-W. 10.1007/s10393-019-01448-w 31664587

[27] Bierque E, Thibeaux R, Girault D, Soupé-Gilbert ME, Goarant C. A systematic review of leptospira in water and soil environments. PLoS ONE. 2020;15:e0227055. 10.1371/JOURNAL.PONE.0227055. 31986154 10.1371/journal.pone.0227055 PMC6984726

[28] Casanovas-Massana A, Pedra GG, Wunder EA, Diggle PJ, Begon M, Ko AI. Quantification of Leptospira interrogans survival in soil and water microcosms. Appl Environ Microbiol. 2018;84. 10.1128/AEM.00507-18. 10.1128/AEM.00507-18 PMC6007094 29703737

[29] Cucchi K, Liu R, Collender PA, Cheng Q, Li C, Hoover CM, et al. Hydroclimatic drivers of highly seasonal leptospirosis Incidence suggest prominent soil reservoir of pathogenic leptospira spp. In rural Western China. PLoS Negl Trop Dis. 2019;13:e0007968. 10.1371/JOURNAL.PNTD.0007968. 31877134 10.1371/journal.pntd.0007968 PMC6948824

[30] Pawar S, Kore M, Athalye A, Thombre P. Seasonality of leptospirosis and its association with rainfall and humidity in Ratnagiri, Maharashtra. Int J Health Allied Sci. 2018;7:37. 10.4103/IJHAS.IJHAS_35_16.

[31] Cunha M, Costa F, Ribeiro GS, Carvalho MS, Reis RB, Nery N, et al. Rainfall and other meteorological factors as drivers of urban transmission of leptospirosis. PLoS Negl Trop Dis. 2022;16:e0007507. 10.1371/JOURNAL.PNTD.0007507. 35404948 10.1371/journal.pntd.0007507 PMC9022820

[32] Sayanthi Y, Susanna D. Pathogenic leptospira contamination in the environment: a systematic review. Infect Ecol Epidemiol. 2024;14. 10.1080/20008686.2024.2324820. 10.1080/20008686.2024.2324820 PMC10953783 38511199

[33] Robertson C, Nelson TA, Stephen C. Spatial epidemiology of suspected clinical leptospirosis in Sri Lanka. Epidemiol Infect. 2012;140:731–43. 10.1017/S0950268811001014. 21676347 10.1017/S0950268811001014

[34] Ehelepola NDB, Ariyaratne K, Dissanayake WP. The correlation between local weather and leptospirosis incidence in Kandy district, Sri Lanka from 2006 to 2015. Glob Health Action. 2019. 10.1080/16549716.2018.1553283. 31154987 10.1080/16549716.2018.1553283 PMC6327921

[35] Gutierrez JD. Effects of meteorological factors on human leptospirosis in Colombia. Int J Biometeorol. 2020;65(2):257–63. 10.1007/S00484-020-02028-2. 10.1007/s00484-020-02028-2 33037904

[36] Syakbanah NL, Fuad A. Human leptospirosis outbreak: A year after the ‘Cempaka’ tropical Cyclone. Jurnal Kesehatan Lingkungan. 2021;13:211–8. 10.20473/JKL.V13I4.2021.211-218.

[37] Tana T, Wada M, Benschop J, Vallee E. The association between rainfall and human leptospirosis in Aotearoa new Zealand. Epidemiol Infect. 2025;153:e112. 10.1017/S0950268825100423. 40855519 10.1017/S0950268825100423 PMC12529419

[38] Denipitiya T, Chandrasekharan V, Abeyewickreme W, Viswakula S, Hapugoda M. Spatial and seasonal analysis of human leptospirosis in the district of Gampaha, Sri Lanka. Sri Lankan J Infect Dis. 2016;6:83. 10.4038/SLJID.V6I2.8109.

[39] Udayanga L, Gunathilaka N, Iqbal MCM, Abeyewickreme W. Climate change induced vulnerability and adaption for dengue incidence in Colombo and Kandy districts: the detailed investigation in Sri Lanka. Infect Dis Poverty. 2020;9:102. 10.1186/S40249-020-00717-Z/FIGURES/9. 32703273 10.1186/s40249-020-00717-z PMC7376859

[40] Jayakody D, Gunathilaka N. Assessing the climate-induced vulnerability and adaptive capacity of cutaneous leishmaniasis diseases in Sri Lanka. In: Jayathilaka N, editor. 24th International Postgraduate Research Conference (IPRC) – 2024. Kelaniya: Faculty of Graduate Studies, University of Kelaniya, Sri Lanka; 2024. p. 51.

[41] Gunathilaka N, Jayakody D. Indicator framework for cutaneous leishmaniasis transmission risk in Sri Lanka using the Delphi-entropy weight method. In: Jayathilaka N, editor. 25th International Postgraduate Research Conference (IPRC) – 2025, Conference Track - Multidisciplinary Studies. Kelaniya: Faculty of Graduate Studies, University of Kelaniya, Sri Lanka; 2025. p. 1.

