# Supplementary material for: Spatiotemporal patterns and climate influences on leptospirosis in Sri Lanka from 2009 to 2024
Source: BMC Infect Dis. 2026 Jan 12;26:159. doi: 10.1186/s12879-026-12533-1 (PMC12838477; doi:10.1186/s12879-026-12533-1)
Supplement: Supplementary file 1 — Supplementary Material 1: 1- Model Comparison [file 12879_2026_12533_MOESM1_ESM.docx]

**Supplementary Material 1- Model Comparison**

The performance of candidate ZAGA‑GAMLSS models was evaluated using Akaike Information Criterion (AIC) and Bayesian Information Criterion (BIC). Table S1 summarizes the fit statistics for five alternative specifications.

**Table S1.** Comparison of candidate ZAGA‑GAMLSS models based on AIC and BIC values

| **Model type** | **AIC** | **BIC** |
| --- | --- | --- |
| Model with all parameters | 12188.73 | 13132.81 |
| Model with all parameters except the minimum and maximum temperature parameters | 12203.05 | 13006.29 |
| Model with lag terms included only in the expected incidence rate component | 12263.9 | 13055.51 |
| Model without lag parameters | 12428.87 | 13025.93 |
| Model without lag parameters and without minimum and maximum temperature | 12446.99 | 13022.02 |

**Justification of Model Selection**

The full model, which included all climatic and spatial predictors along with lag terms, was selected as the final specification. This choice was based on its lowest AIC value (12188.73), indicating a superior fit compared to alternative models. Although the BIC was higher, this reflects BIC’s stronger penalty for model complexity, particularly with large datasets. In epidemiological modeling, explanatory strength and ecological plausibility are prioritized over parsimony alone. All included predictors, temperature, rainfall, humidity, spatial coordinates, and lagged effects, are biologically relevant to leptospirosis transmission. The AIC difference between the full model and the next best alternative exceeded 14 units, which constitutes strong evidence in favor of the more complex model. Thus, the selected model provides the most robust framework for capturing climatic sensitivity, spatial heterogeneity, and temporal autocorrelation in leptospirosis incidence.
